# Intramuscular Hibernoma of the Scapular Region Misdiagnosed on Cytology as a Malignant Lesion: A Report of a Rare Case

**Published:** 2017-10-01

**Authors:** Mukta Pujani, Sabina Khan, Sujata Jetley, Prabhat K Raina

**Affiliations:** 1 *Department of Pathology, ESIC Medical College, Faridabad, India*; 2 *Department of Pathology, Hamdard Institute of Medical Sciences & Research, Jamia Hamdard, New Delhi, India*; 3 *Department of Oncosurgery, RJSP Cancer Hospital, Ranchi, India*

**Keywords:** Hibernoma, Benign Lipomatous Tumor, Brown Fat, Intramuscular Hibernoma, Scapular

## Abstract

Hibernomas are extremely rare benign tumors of adipose tissue characterized by an admixture of brown fat cells with granular, multivacuolated cytoplasm and white fat cells. Hibernomas account for 1.6% of benign adipose tissue tumors and approximately 1.1% of all adipocytic tumors. Around 10% of these cases are intramuscular. It was initially described in the early 1900s as being composed of brown fat. Hibernomas usually occur in third to fourth decades of life and the most frequent sites being thigh, trunk, shoulder, back etc. Cytological differential diagnoses of hibernoma include well differentiated liposarcoma, myxoid/round cell liposarcoma, chondroid lipoma and benign granular cell tumor. Due to its abundant vascularity evident on angiography, it can sometimes mimic a malignant lesion, from which it needs to be distinguished as complete surgical excision is the only treatment required for a hibernoma. Moreover, it has no malignant or metastatic potential.

We presented a rare case of intramuscular hibernoma of the scapular region in a 34-year-old male, in which cytology was reported as pleomorphic fibrolipomatous malignant lesion.

## Introduction

Hibernoma is a rare benign tumor of adipose tissue, which is composed of brown fat cells with granular, multivacuolated cytoplasm at least in part. This component may be admixed with white adipose tissue in variable proportions. It accounts for 1.1% of all adipocytic tumors. The most common site for hibernoma is thigh, followed by trunk, upper extremity and head and neck. Around 10% of cases are intramuscular (1, 2, 3).

We presented a rare case of intramuscular hibernoma of the scapular region in a 34-year-old male, in whom cytology from a private laboratory was reported as pleomorphic fibrolipomatous malignant lesion.

## Case Summary

A 34-year-old male presented to the surgery clinics of Hakeem Abdul Hameed Centenary hospital with a painless progressive swelling in the left scapular region for the last two months. The lump was slowly increasing in size, but not associated with backache, shoulder movement restriction, cough, chest pain or dyspnea. There was no history of any other swelling in the body, any chronic medical illness or any prior surgery.

On examination, vital signs were stable. Systemic examination was within the normal limits. There was no lymphadenopathy. Local examination revealed a diffuse soft swelling in the left scapular and infrascapular region with ill-defined margins. Skin over the lump had normal temperature and was non-tender. A clinical diagnosis of soft tissue tumor arising from the muscle was considered.

Fine needle aspiration cytology (FNAC) of the lump had been performed in a private laboratory before the patient came to our hospital. According to the report, smears showed tumor cells scattered and distributed in fragments of fibromyxoid tissue. Highly atypical lipoblasts and small sized pleomorphic tumor cells were also seen. Findings were suggestive of a pleomorphic fibrolipo malignant lesion. 

**Figure 1:**
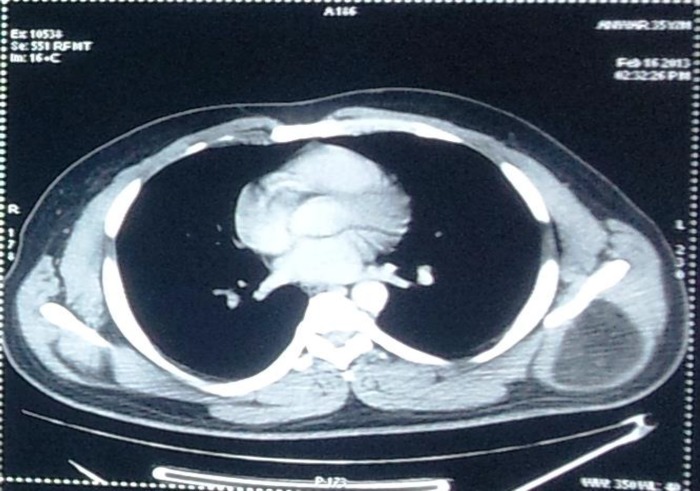
CECT shows a well circumscribed, minimally enhancing, hypodense lesion measuring 8x7.5cm with attenuation corresponding to fat density in left periscapular area

Based on FNAC findings, contrast enhanced computed tomography (CECT) scan was performed which revealed a minimally enhancing well-defined circumscribed oval shaped fat attenuation lesion measuring 6.5x5x11.4 cm in left periscapular area, abutting and involving periscapular muscles (Figure 1).

No evidence of scapular erosion or extension into the overlying skin and subcutaneous tissue was noted.

**Figure 2:**
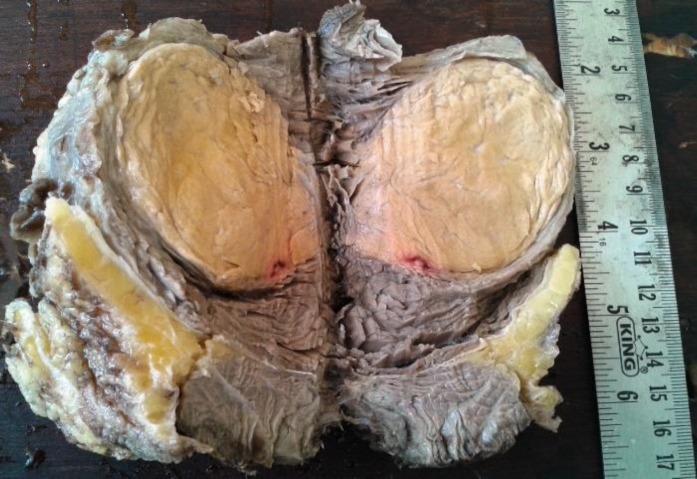
Gross specimen shows a well defined, encapsulated, intramuscular tumor with a yellowish brown cut surface

Wide local excision was performed and specimen sent for histopathology. On gross examination, tumor was well circumscribed, encapsulated and intramuscularly located measuring 7x6x5cm with a brownish yellow cut surface (figure 2). 

Overlying skin and surrounding subcutaneous tissue and muscle appeared to be free of tumor. Tumor was around 0.5cm away from deep resection margin.

**Figure 3:**
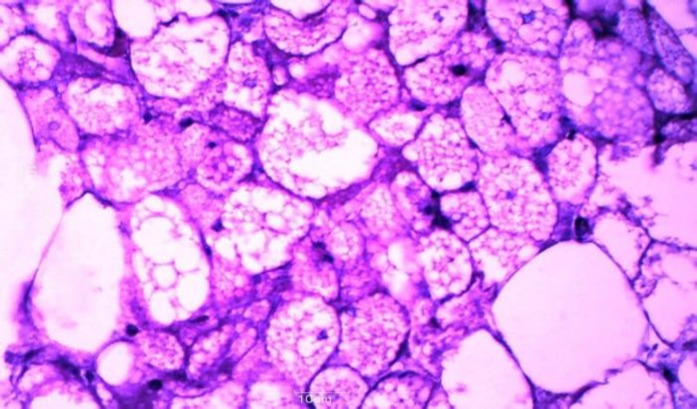
Microscopic section showed an admixture of multivacuolated, granular cells and univacuolated mature adipocytes (Hematoxylin and Eosin, 400X

On histopathology, Hematoxylin and Eosin stained sections showed a well encapsulated, intramuscular tumor composed of large number of cells with abundant, granular multivacuolated cytoplasm and a small central nucleus with a single prominent nucleolus in some cells. Few pale staining cells and univacuolated mature white adipose tissue cells were also seen (figure 3). 

There was no evidence of cellular atypia or mitosis. No areas of myxoid change or spindle cell component seen. There was no infiltration into subcutaneous tissue or surrounding muscles. A final diagnosis of hibernoma, typical variant was rendered.

## Discussion

Hibernomas are extremely rare benign tumors of adipose tissue characterized by an admixture of brown fat cells with granular, multivacuolated cytoplasm and white fat cells. They were previously diagnosed as adenoma of sebaceous gland, pseudolipoma or lipomas of immature adipose tissue (4).

Hibernomas account for 1.6% of benign adipose tissue tumors and approximately 1.1% of all adipocytic tumors (1). Merkel was the first to describe it as a tumor of brown adipose tissue way back in 1906 (4). However, the term hibernoma was first described by Gery in 1914 due to its resemblance to the cells so called hibernating glands of animals (5). The etiology of hibernoma, however, remains unknown.

Brown adipose tissue is thought to have a thermoregulatory function and it is first seen at 21^st^ week of gestation, in the human fetus. In adults, brown fat is confined to the more central parts of the body leading to vest like distribution in the neck, axilla, mediastinum and periadrenal areas. 

 Unlike other adipose tissue tumors which occur in 5^th^ to 7^th^ decades, hibernomas usually occur in the third decade. Furlong et al. conducted a clinicopathological study of 170 cases of hibernoma and found that the mean age was 38 years, ranging from 2 to 75 years (2). The most common location of hibernoma was thigh (n=50 cases), followed by shoulder (n=20 cases), back (n=17 cases), neck (n=16 cases), chest (n=11 cases), arm (n=11 cases) and abdominal cavity/ retroperitoneum (n=10 cases). In a review of 17 cases of hibernomas by Mavrogenis et al. the most common location was found to be thigh, followed by buttock and scapula (3). We came across a few reports in the interscapular region similar to our case (6, 7)

The tumor size of hibernomas may range from 1 to 24 cm with an average diameter of 9.3 cm. A few rare case reports of massive hibernomas have been reported in abdominal wall and neck (8, 9). Hibernomas are well encapsulated tumors with a brownish yellow cut surface without infiltration into the surrounding structures.

Cytologically, the smears usually show clusters of uniform round cells with cytoplasmic vacuoles with central round nuclei and evenly distributed chromatin and small nucleoli. The cytological differential diagnoses of hibernoma include well differentiated liposarcoma, myxoid/round cell liposarcoma, chondroid lipoma and benign granular cell tumor. Presence of univacuolated or multivacuolated fat cells, rich, delicate capillary like vasculature and large stripped nuclei with distinct nucleoli may lead to a suspicion of liposarcoma (10, 11) 

Histopathologically, four morphological variants of hibernoma have been identified, based on the type of hibernoma cells, stroma and presence of spindle cell component. The most common type is typical hibernoma which accounts for 82% of all cases of hibernoma and is characterized by an admixture of eosinophilic cells, hibernoma cells and white fat cells. 

The second most common type, myxoid variant (9% cases), is composed of multivacuolated cells with focal eosinophilic cytoplasm contained in a loose basophilic matrix. The lipoma-like variant (7% cases) is composed of univacuolated mature fat cells with only scattered hibernoma cells. Tumors with multivacuolated adipose cells more than or equal to 70% are labelled as typical hibernomas (non-lipoma hibernomas), while those with less than 70% multivacuolated cells are considered as lipoma-like hibernoma.

The least common type is the spindle cell variant (2% cases). It shows features overlapping hibernomas and spindle cell lipomas and is characterized by multivacuolated cells seen in hibernoma in addition to adipocytes, spindle cells, mast cells and collagen bundles. Despite this morphological classification, all these variants have the same good prognosis following complete excision (1-3, 12). The present case was an example of a typical hibernoma.

There is usually no cytological atypia and mitosis is also not a feature of hibernoma. In this case, the multivacuolated cells with central nuclei and occasional prominent nucleoli were probably mistaken as lipoblasts on FNAC. However, absence of mitosis in these cells was a clue to the benign nature of the lesion that was overlooked at that time. 

Immunophenotypically, hibernomas usually show positive results for S-100. The spindle cell variant is typically CD34 positive.

In a case of hibernoma, radiographs do not show any calcifications or bone erosion. Ultrasonography of a hibernoma reveals a well-circumscribed uniformly hyperechoic mass. On CT scan, the lesion is usually lobulated, septated and well circumscribed with fat density and variable contrast enhancement. On T_1_ weighted MRI scans, hibernomas appear hypointense to fat while on T_2_ images they are usually isointense to subcutaneous fat. Radiological and histological differential diagnoses of hibernomas include other benign soft tissue tumors like lipomas, rhabdomyomas, neurofibromas, fibroma, hemangiomas as well as malignant tumors including well differentiated liposarcomas or metastatic carcinoma (13, 14)

Angiography of hibernomas reveals rich vascularity and sometimes arteriovenous shunting. However, angiography can sometimes mislead surgeons towards a malignant process. 

In hibernomas, the only recurrent aberration is involvement of 11q13-21 in structural rearrangements, which usually affect three or more chromosomes. Complete local excision is the treatment of choice for hibernomas as recurrences have not been reported (1).

## Conflict of Interest

The authors have no ﬁnancial relationships with any organization.
